# ER Stress and Micronuclei Cluster: Stress Response Contributes to Genome Chaos in Cancer

**DOI:** 10.3389/fcell.2021.673188

**Published:** 2021-08-04

**Authors:** Eric Heng, Amanda Moy, Guo Liu, Henry H. Heng, Kezhong Zhang

**Affiliations:** Center for Molecular Medicine and Genetics, Wayne State University School of Medicine, Detroit, MI, United States

**Keywords:** cancer evolution, chromosomal instability, karyotype coding, non-clonal chromosome aberrations, polyploid giant cancer cells, two-phased cancer evolution, genome architecture theory, system inheritance

## Background

Micronuclei is a cytogenetic term describing small nuclei composed of one to a few chromosomes or chromosomal fragments, which often result from abnormal cell divisions (Fenech et al., 2016). Micronuclei were discovered by Henry Howell and Justin Marie Jolly in erythrocytes over a century ago (Sears and Udden, 2012). Initially, Howell-Jolly bodies were used to describe the visible DNA fragments following the expulsion of the nucleus. Later on, the term “micronuclei” was adapted to describe all smaller fragments of the nucleus (1/5−1/20 the size of a normal nucleus) (Evans et al., 1959; Schmid, 1975). Micronuclei were initially correlated with conditions such as vitamin B12 and folate deficiency (Dawson and Bury, 1961). Soon thereafter, they were linked to many other genotoxic factors such as chemical exposure and radiation (Fenech et al., 2011). The presence of micronuclei is usually considered as an indicator of DNA damage and defects in mitosis.

Several different assays have been developed for the use of micronuclei. The cytokinesis-block, peripheral lymphocytic, and buccal MN assays have all been developed for use in mammalian cells *in vivo* (Sommer et al., 2020). Particularly, the development of the peripheral lymphocytic MN assay pioneered the use of micronuclei as a biomarker in mammalian cells with higher efficiency, along with a more rigorous definition for scoring (<1/3 of a nucleus, similar staining, no overlap, within 3–4 nuclear diameters from the main nucleus) (Countryman and Heddle, 1976).

Recently, enthusiasm for micronuclei studies has reemerged (Xie et al., 2016; Guo et al., 2019, 2020a,b; Fenech, 2020; Lepage et al., 2020; Mirzayans and Murray, 2020). First, the linkage to chromosomal instability, cancer, and other aging-related diseases has made micronuclei a potential potent biomarker (Aranda et al., 2018). Secondly, micronuclei, among many other previously ignored chromosomal abnormalities (most of which belong to the non-clonal chromosome aberrations or NCCAs), are key contributing factors for cancer by re-organizing the karyotype coding (Iourov et al., 2010, 2020; Heng et al., 2013; Ye et al., 2019a). Thirdly, micronuclei involve the activation of the immune system, further broadening the utility of micronuclei (Bartsch et al., 2017; Mackenzie et al., 2017; Kirsch-Volders et al., 2020). Finally, micronuclei are linked to stress response-mediated genome chaos, a driving force for cancer evolution (Heng, 2019; Ye et al., 2019b, 2021; Shoshani et al., 2020).

## Micronuclei Clusters: Changing the System Inheritance

**Figure 1:**
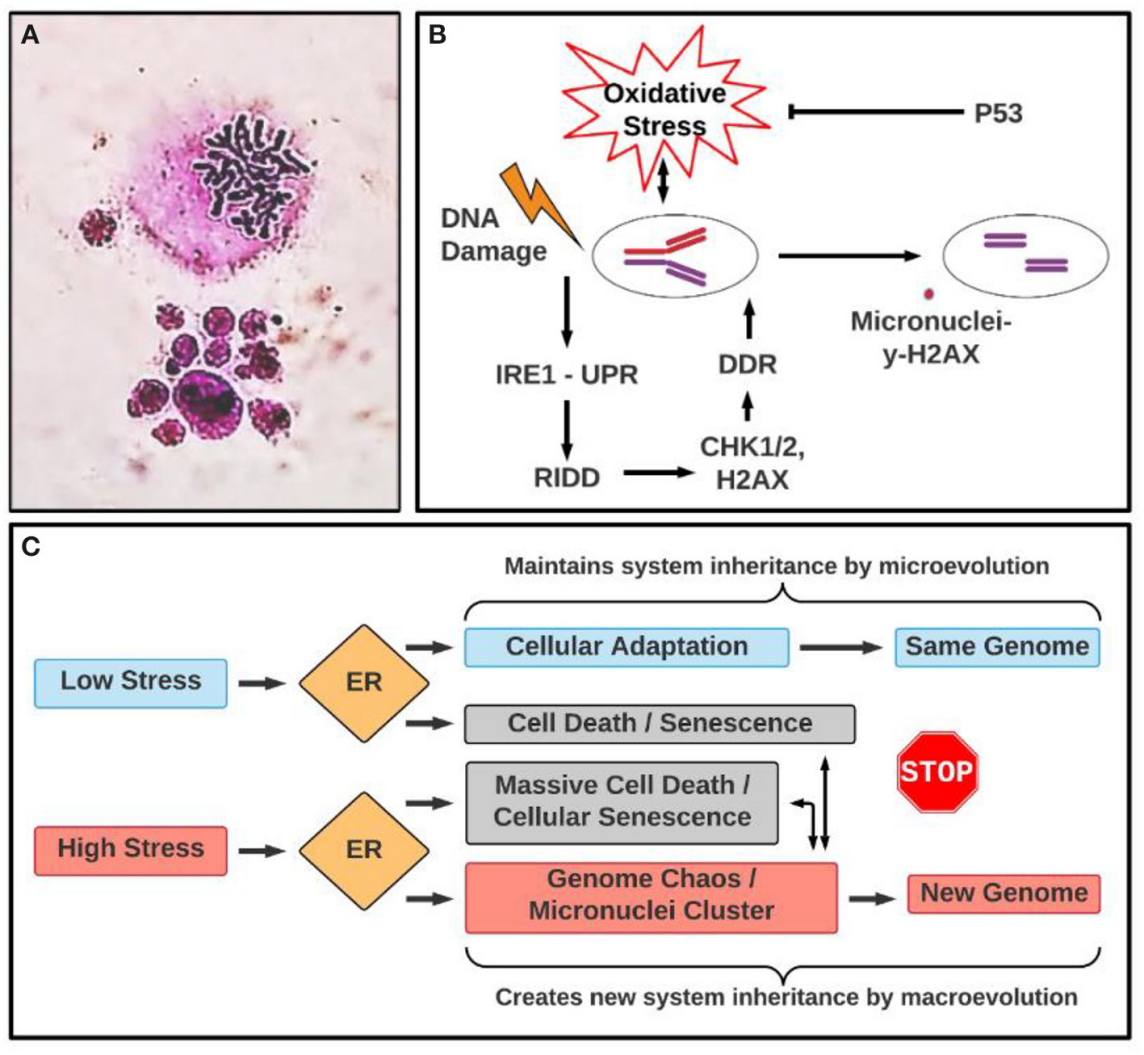
Micronuclei cluster: morphology and involved pathways in ER stress. **(A)** Example of micronuclei cluster. The upper image is a mitotic figure and the lower image is a micronuclei cluster where different sized nuclei clustered with each other. Mitotic figure and micronuclei cluster was stained by Giemsa. **(B)** Illustrating a link between oxidative stress, unfolded protein response (UPR) and micronuclei formation under genotoxic stress. DDR, DNA damage response; RIDD: IRE1α-dependent decay. **(C)** A proposed conceptual relationship between different types of stresses, ER stress response, the types of evolution (macroevolution vs. microevolution). Even though ER response acts differently according to low and high stress, which are linked to microevolution and macroevolution, respectively, they are overlapping in terms of leading to senescence and genome chaos. For example, while high stress often can induce genome chaos including micronuclei clusters and senescence, coupling with massive death, low stress also could lead to death and senescence, which can lead to genome reorganization, albeit at a much lower rate. The association between senescence and ER stress response (UPR) is context-dependent. Indeed, senescence and ER stress response (UPR) make an interconnected network (UPR is activated in consequence to cell senescence or UPR is a driver of senescence) in which oxidative stress (ROS) acts as a central element responsible for an auto-amplification loop (Pluquet et al., 2015). The conceptual basis of this model is stress-induced genome reorganization that is essential for somatic evolution. Cellular stress in general, and ER stress in particular, can serve as an informational code that determines the cell's fate (Heng and Heng, 2021). Depending on the stress intensity or duration, UPR can trigger diverse cellular responses, including apoptotic pathways, which can be linked to genome chaos with the involvement of micronuclei clusters. This model also predicts the complex relationship between the UPR, genome reorganization, and functional relevance in cancer immunobalance. Even though the UPR may promote genome reorganization under acute or severe stress conditions, newly formed genomes can either be favorable or unfavorable for cancer cell malignancy depending on the genomic context. For example, the newly formed genomes, including micronuclei clusters, could either activate the immune system to fight against cancer or instead help cancer unexpectedly. It was reported that cell fusion can occur between cancer cells and immune cells (such as macrophages), promoting cancer cells to become more aggressive (Gast et al., 2018). In fact, a German gynecologist, Otto Aichel, first introduced this idea in 1911. It is likely that fused hybrid cells change their genomes through genome chaos including micronuclei clusters. According to the Genome Architecture Theory, such a mechanism of genome information creation under stress should be a universal phenomenon. However, for future research direction, quantitative studies are needed to predict the clinical odds (beneficial or harmful) under different treatment conditions.

Micronuclei clusters are a group of micronuclei with variable size, which are often generated from one cell (either diploid or polyploid cell) (Figure 1A). These structures have been largely ignored, based on the reasoning that they represented cells that would soon be dead regardless. However, recent studies have demonstrated that micronuclei clusters belong to a type of chaotic genome, some of which can continue to evolve by cellular fusion and fission, representing important transitional structures for cellular macroevolution (Heng, 2015, 2019). In fact, the micronuclei cluster is most commonly observed in cancer samples, especially following drug treatment (Heng et al., 2008). Moreover, polyploid giant cancer cells (PGCCs) have generated excitement in cancer research lately, due to their contribution to rapid drug resistance and induced cancer lethality (Mirzayans et al., 2018; Erenpreisa et al., 2020; Mannan et al., 2020; Pienta et al., 2020a,b). PGCCs belong to numerical genome chaos, which can generate many aggressive near-diploid cancer cells with newly rearranged karyotypes (Heng et al., 2008; Liu et al., 2014). During this transition, PGCCs also can generate polyploid types of micronuclei clusters (Heng et al., 2013; Zhang et al., 2014). A model of how PGCCs contribute to drug resistance and aggressive cancer growth has been proposed, which involves high levels of cellular stress-induced abnormal developmental processes (dedifferentiation), genome chaos-mediated macroevolution (creation of new genome systems by PGCCs in smaller cell populations), and microevolution (growth of stable cancer populations) (Niu et al., 2016, 2017; Liu, 2018, 2020). This series of studies has also highlighted the importance of micronuclei, as they have now been shown to be initiators of genome instability and macroevolution, rather than just a reflection of genotoxic conditions. Along these lines, micronuclei clusters are often detected from drug treatment-induced genome chaos, including PGCCs. Clearly, the micronuclei cluster represents a means to change the karyotype coding, and by extension, the system inheritance (Ye et al., 2019b).

## Search For The Link Between Endoplasmic Reticulum (ER) Stress, Micronuclei, and Giant Cells

Interestingly, the cellular stress response machinery controls both the switching of genome stability and the dynamics leading to a spectrum of numerical and structural karyotypic variants (normal to chaotic) (Beaupere and Labunskyy, 2019; Heng, 2019; Limia et al., 2019). It is thus necessary to investigate how ER stress response impacts micronuclei and their triggered genome chaos (Heng et al., 2013; Zhang et al., 2015). The ER is responsible for the majority of cellular protein synthesis and folding while playing a key role in sensing cellular stress. For example, ER stress response or unfolded protein response (UPR) can either promote cellular survival or commit the cell to a pathway of apoptosis under different stress conditions. There are already two types of established linkages between intracellular stress and micronuclei: (1). Linking ER stress and oxidative stress responses to micronuclei/chromosome instability; (2). Linking ER stress to micronuclei clusters and polyploidy.

Most publications belong to the first category. For example, many studies suggest that micronuclei not only serve as an index of genotoxic effects and chromosomal instability (Guo et al., 2020b), but also are associated with the cellular stress response and immune activation following DNA damage (Chatterjee et al., 2018). Many biochemical and pathophysiological conditions, such as double-stranded DNA breaks, impaired DNA repair response, improper DNA replication, treatment of DNA adduct-forming chemicals, inhibition of microtubule polymerization, and centromere interference (Ye et al., 2019b; Guo et al., 2020a) can all directly or indirectly cause the formation of micronuclei. Studies further suggest that micronuclei can result from natural processes, such as metabolism and aging, and can be induced by environmental factors or irregular lifestyles (Luzhna et al., 2013; Nefic and Handzic, 2013). Over the past decade, mounting evidence suggests that micronucleus-causing genotoxicity is intrinsically linked to intracellular stress responses, particularly oxidative stress and ER stress responses (Luzhna et al., 2013; Horne et al., 2014; Hetz et al., 2020).

During mitotic exit, mis-segregated chromosomes can recruit their own nuclear envelope to form micronuclei. Over 60% of micronuclei undergo an irreversible loss of compartmentalization during interphase due to the collapse of the nuclear envelope (Hatch et al., 2013). This disruption of micronuclei reduces nuclear functions and can trigger massive DNA damage. Micronuclei disruption is associated with chromatin compaction and invasion of ER tubules into the chromatin. Disrupted micronuclei were identified in both major subtypes of human non-small cell lung cancer, solidifying evidence that disrupted micronuclei are useful objective biomarkers for genomic instability in cancer.

The process leading to the formation of micronuclei is associated with intracellular stress response and genotoxicity. It has been demonstrated that oxidative stress preferentially induces a subtype of micronuclei, specifically, the subclass marked by pan-staining of γ-H2AX or γ-H2AX (+), and mediates genomic instability caused by p53 dysfunction (Xu et al., 2014) (Figure 1B). Furthermore, reactive oxygen species (ROS) are known to cause many types of DNA lesions that can be converted into cancer-promoting genetic alterations. However, the tumor suppressor p53 plays an important role in regulating the generation of cellular ROS, by reducing oxidative stress under physiological or stress conditions. Indeed, in human and mouse cells that are deficient in p53, the frequencies of γ-H2AX (+) micronuclei are significantly elevated but can be attenuated by the antioxidant N-acetylcysteine (NAC). These findings implicate the importance of p53-regulated redox levels in the maintenance of genomic stability by preventing the formation of micronuclei.

Genotoxic stress-induced micronuclei formation is associated with the activation of ER stress response or UPR that modulates DNA damage repair programs and sustains cell survival (Dufey et al., 2020). DNA damage triggers the UPR signaling pathway mediated through the ER stress sensor inositol-requiring enzyme 1α (IRE1α), leading to the activation of regulated IRE1α-dependent decay (RIDD). The RIDD pathway sustains the activity of the key factors involved in the DNA damage response, including checkpoint kinase (CHK) 1, CHK2, and H2A.X Variant Histone (H2AX), therefore boosting the DNA damage response (DDR) (Figure 1B) (Dufey et al., 2020). Through modulation of DNA damage repair, cell cycle arrest, and apoptosis, UPR signaling through IRE1α may preserve genome stability and therefore protect the disruption of micronuclei under genotoxic stress. Additionally, ER stress and inflammatory responses are linked to genomic instability induced by gamma radiation (Chatterjee et al., 2018).

As of late, an increasing number of reports belonging to the second category that links ER stress to micronuclei clusters and polyploidy are being produced. Micronuclei clusters have been frequently observed in cancer, but many images of them were left unpublished due to their supposed lack of scientific worth (Hatch et al., 2013; Ye et al., 2019b). As the interaction of PGCCs and genome chaos-mediated macroevolution represents a newly emerging field in cancer research, we anticipate many studies of the same type to soon follow (Heng and Heng, 2020; Ye et al., 2021).

ER stress response has been linked to drug-induced-chromosome fragmentation, a type of mitotic cell death, and micronuclei clusters (Stevens et al., 2007, 2011). Similarly, ER stress response has been linked to chromosome instability in GWI (unpublished observation). Another noted study used the ER stress response to study mechanisms of genomic instability in polyploidization. Ploidy can activate ER stress response, resulting in anti-cancer immune responses. Immunoselection can then reduce ploidy, ER stress, and calreticulin exposure (Senovilla et al., 2012, 2017). It is thus interesting to study the complex relationship between ER stress, PGCCs, micronuclei clusters, and immune responses, which may yield new discoveries.

## Future Perspective

The ER stress response represents a major regulating mechanism for cellular function under stress. Since both ER stress and micronuclei clusters can be linked to numerical molecular pathways, and the micronuclei are linked to genome chaos (Zhang et al., 2015; Ye et al., 2019b), we expect a sizable number of diverse molecular mechanisms to be published in the near future. It is thus essential to research the common mechanisms rather than focus on specific, individual ones, as in complex systems, there will always be too many genomic and environmental factors involved, with most contributing only moderately. Since CIN can be used to unify the triggering factors of cancer evolution including drug resistance (Ye et al., 2020, 2021), establishing the quantitative link between the ER stress response and CIN is a promising starting point. The quantitative data of micronuclei, especially of micronuclei clusters, is of importance when integrating into this platform.

Depending on the context, the ER stress response can play paradoxical roles. Under normal circumstances, it maintains system stability by repairing and eliminating unrepairable cellular elements or cells; under crisis, it might promote changes by incomplete cell death, and genome chaos (Heng, 2015, 2019). It is known that during cellular death, some outliers can form survivable genomes, and form new cellular populations. Furthermore, according to the Genome Architecture Theory, karyotype coding is maintained via the function of sexual reproduction and somatic genome instability (Gorelick and Heng, 2011; Ye et al., 2019a). Under crisis, however, the process of genome chaos can create new karyotype coding systems for speciation. It is thus timely to investigate the role played by ER stress response in this process. Similar opposing functions can be found in cancer immune surveillance as well. As mentioned previously, while ploidy can activate anti-cancer immune responses via the ER stress response (Senovilla et al., 2012), a high level of chromosomal structural variations can also suppress the immune response to cancer (Minton, 2012; Zanetti and Mahadevan, 2012; Zanetti, 2017). In other words, chromosomal chaos may promote or silence immune surveillance depending on different environmental and informational contexts. Consequently, how ER stress plays a role in the interplay between chromosomal abnormalities and immune surveillance is of importance in cancer research.

During tumorigenesis, high proliferation rates of cancer cells demand increased activities of ER protein folding and transport, a condition that triggers ER stress. As tumors grow, cancer cells experience nutrient starvation and hypoxia, which can induce the accumulation of unfolded or misfolded proteins in the ER and activation of the ER stress response (Hetz et al., 2020). It has been demonstrated that ER stress response is an important mechanism required for cancer cells to adapt to and survive from oncogenic stress conditions (Wang et al., 2010). Recently, the cancer problem, traditionally considered as an issue of out-of-control growth, has been rephrased as different phase transitions (from normal cells to transformed cells, from non-invasive tumor to cancer, and from drug-sensitive cancer cells to drug-resistant cells) (Heng and Heng, 2020). Accordingly, it would be interesting to investigate the ER homeostasis during these various phase transitions. Such types of information, including different ER stress response pathways, levels of overall stress, and types of genomic information involved (gene, epigenetic, and karyotype changes), are essential to understand the stress-information relationship in somatic and organismal evolution (Kültz, 2005, 2020; Heng, 2019). To initiate such an effort, a model that illustrates the relationship between stress levels, cellular responses, and types of evolution is proposed for future study (Figure 1C).

## Author Contributions

EH, KZ, and HH drafted the manuscript. AM and GL participated in the initial observations and discussion. AM was a Summer Undergraduate Research Student of Center for Molecular Medicine and Genomics, Wayne State University. EH was a summer student. All authors involved in literature search and editing of the manuscript.

## Conflict of Interest

The authors declare that the research was conducted in the absence of any commercial or financial relationships that could be construed as a potential conflict of interest.

## Publisher's Note

All claims expressed in this article are solely those of the authors and do not necessarily represent those of their affiliated organizations, or those of the publisher, the editors and the reviewers. Any product that may be evaluated in this article, or claim that may be made by its manufacturer, is not guaranteed or endorsed by the publisher.

## References

[B1] Aranda F. Chaba K. Bloy N. Garcia P. Bordenave C. Martins I.. (2018). Immune effectors responsible for the elimination of hyperploid cancer cells. Oncoimmunology 7:e1463947. 10.1080/2162402X.2018.1463947 30221060 PMC6136857

[B2] Bartsch K. Knittler K. Borowski C. Rudnik S. Damme M. Aden K.. (2017). Absence of RNase H2 triggers generation of immunogenic micronuclei removed by autophagy. Hum. Mol. Genet. 26, 3960–3972. 10.1093/hmg/ddx283 29016854

[B3] Beaupere C. Labunskyy V. M. (2019). (Un)folding mechanisms of adaptation to ER stress: lessons from aneuploidy. Curr. Genet. 65, 467–471. 10.1007/s00294-018-0914-9 30511161 PMC6421085

[B4] Chatterjee J. Nairy R. K. Langhnoja J. Tripathi A. Patil R. K. Pillai P. P.. (2018). ER stress and genomic instability induced by gamma radiation in mice primary cultured glial cells. Metab Brain Dis. 33, 855–868. 10.1007/s11011-018-0183-9 29429012

[B5] Countryman P. I. Heddle J. A. (1976). The production of micronuclei from chromosome aberrations in irradiated cultures of human lymphocytes. Mutat. Res. 41, 321–332. 10.1016/0027-5107(76)90105-6 796719

[B6] Dawson D. W. Bury H. P. (1961). The significance of Howell-Jolly bodies and giant metamyelocytes in marrow smears. J. Clin. Pathol. 14, 374–380. 10.1136/jcp.14.4.374 13720318 PMC480237

[B7] Dufey E. Bravo-San Pedro J. M. Eggers C. González-Quiroz M. Urra H. Sagredo A. I.. (2020). Genotoxic stress triggers the activation of IRE1α-dependent RNA decay to modulate the DNA damage response. Nat. Commun. 11:2401. 10.1038/s41467-020-15694-y 32409639 PMC7224204

[B8] Erenpreisa J. Salmina K. Anatskaya O. Cragg M. S. (2020). Paradoxes of cancer: survival at the brink. Semin. Cancer Biol. 10.1016/j.semcancer.2020.12.009. [Epub ahead of print]. 33340646

[B9] Evans H. J. Neary G. J. Williamson F. S. (1959) The relative biological efficiency of single doses of fast neutrons gamma-rays on vicia faba roots the effect of oxygen. Int. J. Radiat. Biol. Relat. Stud. Phys. Chem. Med. 3, 216–229. 10.1080/09553005914550311 13820987

[B10] Fenech M. (2020). Cytokinesis-block micronucleus cytome assay evolution into a more comprehensive method to measure chromosomal instability. Genes. 11:1203. 10.3390/genes11101203 33076531 PMC7602810

[B11] Fenech M. Kirsch-Volders M. Natarajan A. T. Surralles J. Crott J. W. Parry J.. (2011). Molecular mechanisms of micronucleus, nucleoplasmic bridge and nuclear bud formation in mammalian and human cells. Mutagenesis 26, 125–132. 10.1093/mutage/geq052 21164193

[B12] Fenech M. Knasmueller S. Bolognesi C. Bonassi S. Holland N. Migliore L.. (2016). Molecular mechanisms by which *in vivo* exposure to exogenous chemical genotoxic agents can lead to micronucleus formation in lymphocytes *in vivo* and *ex vivo* in humans. Mutat Res. 770, 12–25. 10.1016/j.mrrev.2016.04.008 27894682

[B13] Gast C. E. Silk A. D. Zarour L. Riegler L. Burkhart J. G. Gustafson K. T.. (2018). Cell fusion potentiates tumor heterogeneity and reveals circulating hybrid cells that correlate with stage and survival. Sci Adv. 4:eaat7828. 10.1126/sciadv.aat7828 30214939 PMC6135550

[B14] Gorelick R. Heng H. H. (2011). Sex reduces genetic variation: a multidisciplinary review. Evolution 65, 1088–1098. 10.1111/j.1558-5646.2010.01173.x 21091466

[B15] Guo X. Dai X. Wu X. Zhou T. Ni J. Xue J.. (2020b). Understanding the birth of rupture-prone and irreparable micronuclei. Chromosoma 129, 181–200. 10.1007/s00412-020-00741-w 32671520

[B16] Guo X. Dai X. Zhou T. Wang H. Ni J. Xue J.. (2020a). Mosaic loss of human Y chromosome: what, how and why. Human Genet. 139, 421–446. 10.1007/s00439-020-02114-w 32020362

[B17] Guo X. Ni J. Liang Z. Xue J. Fenech M. F. Wang X. (2019). The molecular origins and pathophysiological consequences of micronuclei: new insights into an age-old problem. Mutat. Res. 779, 1–35. 10.1016/j.mrrev.2018.11.001 31097147

[B18] Hatch E. M. Fischer A. H. Deerinck T. J. Hetzer M. W. (2013). Catastrophic nuclear envelope collapse in cancer cell micronuclei. Cell 154, 47–60. 10.1016/j.cell.2013.06.007 23827674 PMC3749778

[B19] Heng H. H. (2015). Debating Cancer: The Paradox in Cancer Research. Singapore: World Scientific Publishing Co. ISBN 978-981-4520-84-3. 10.1142/8879

[B20] Heng H. H. (2019). Genome Chaos: Rethinking Genetics, Evolution, and Molecular Medicine. Cambridge, MA: Academic Press Elsevier. ISBN 978-012-8136-35-5.

[B21] Heng H. H. Liu G. Stevens J. B. Abdallah B. Y. Horne S. D. Ye K. J.. (2013). Karyotype heterogeneity and unclassified chromosomal abnormalities. Cytogenet. Genome Res. 139, 144–157. 10.1159/000348682 23571381

[B22] Heng H. H. Stevens J. B. Lawrenson L. Liu G. Ye K. J. Bremer S. W.. (2008). Patterns of genome dynamics and cancer evolution. Cell. Oncol. 30, 513–514. 10.1155/2008/267326 18936532 PMC4618818

[B23] Heng J. Heng H. H. (2020). Genome chaos: creating new genomic information essential for cancer macroevolution. Semin. Cancer Biol. 10.1016/j.semcancer.2020.11.003. [Epub ahead of print]. 33189848

[B24] Heng J. Heng H. H. (2021). Karyotype coding: the creation and maintenance of system information for complexity and biodiversity. Biosystem 208:104476. 10.1016/j.biosystems.2021.104476 34237348

[B25] Hetz C. Zhang K. Kaufman R. J. (2020). Mechanisms, regulation and functions of the unfolded protein response. Nat. Rev. Mol. Cell Biol. 21, 421–438. 10.1038/s41580-020-0250-z 32457508 PMC8867924

[B26] Horne S. D. Chowdhury S. K. Heng H. H. (2014). Stress, genomic adaptation, and the evolutionary trade-off. Front. Genet. 5:92. 10.3389/fgene.2014.00092 24795754 PMC4005935

[B27] Iourov I. Y. Vorsanova S. G. Yurov Y. B. (2010). Somatic genome variations in health and disease. Curr. Genomics 11, 387–396. 10.2174/138920210793176065 21358982 PMC3018718

[B28] Iourov I. Y. Vorsanova S. G. Yurov Y. B. Zelenova M. A. Kurinnaia O. S. Vasin K. S.. (2020). The cytogenomic “Theory of Everything”: chromohelkosis may underlie chromosomal instability and mosaicism in disease and aging. Int. J. Mol. Sci. 21:8328. 10.3390/ijms21218328 33171981 PMC7664247

[B29] Kirsch-Volders M. Bolognesi C. Ceppi M. Bruzzone M. Fenech M. (2020). Micronuclei, inflammation and auto-immune disease. Mutat. Res. 786:108335. 10.1016/j.mrrev.2020.108335 33339583

[B30] Kültz D. (2005). Molecular and evolutionary basis of the cellular stress response. Annu. Rev. Physiol. 67, 225–257. 10.1146/annurev.physiol.67.040403.103635 15709958

[B31] Kültz D. (2020). Evolution of cellular stress response mechanisms. J. Exp. Zool. A Ecol. Integrative Physiol. 333, 359–378. 10.1002/jez.2347 31970941

[B32] Lepage C. C. Thompson L. L. Larson B. McManus K. J. (2020). An automated, single cell quantitative imaging microscopy approach to assess micronucleus formation, genotoxicity and chromosome instability. Cells 9:344. 10.3390/cells9020344 32024251 PMC7072510

[B33] Limia C. M. Sauzay C. Urra H. Hetz C. Chevet E. Avril T. (2019). Emerging roles of the endoplasmic reticulum associated unfolded protein response in cancer cell migration and invasion. Cancers 11:631. 10.3390/cancers11050631 31064137 PMC6562633

[B34] Liu G. Stevens J. Horne S. Abdallah B. Y. Ye K. J. Bremer S. W.. (2014). Genome chaos: Survival strategy during crisis. Cell Cycle 13, 528–537. 10.4161/cc.27378 24299711 PMC6093293

[B35] Liu J. (2018). The dualistic origin of human tumors. Semin. Cancer Biol. 53, 1–16. 10.1016/j.semcancer.2018.07.004 30040989 PMC6553492

[B36] Liu J. (2020). The “life code”: a theory that unifies the human life cycle and the origin of human tumors. Semin. Cancer Biol. 60, 380–397. 10.1016/j.semcancer.2019.09.005 31521747 PMC13217516

[B37] Luzhna L. Kathiria P. Kovalchuk O. (2013). Micronuclei in genotoxicity assessment: from genetics to epigenetics and beyond. Front. Genet. 4:131. 10.3389/fgene.2013.00131 23874352 PMC3708156

[B38] Mackenzie K. J. Carroll P. Martin C. A. Murina O. Fluteau A. Simpson D. J.. (2017). cGAS surveillance of micronuclei links genome instability to innate immunity. Nature 548, 461–465. 10.1038/nature23449 28738408 PMC5870830

[B39] Mannan R. Wang X. Bawa P. S. Spratt D. E. Wilson A. Jentzen J.. (2020). Polypoidal giant cancer cells in metastatic castration-resistant prostate cancer: observations from the Michigan Legacy Tissue Program. Med. Oncol. 37:16. 10.1007/s12032-020-1341-6 32030484 PMC8208238

[B40] Minton K. (2012). Tumour immunology: chromosome overload. Nat. Rev. Immunol. 12:745. 10.1038/nri3326 23037554

[B41] Mirzayans R. Andrais B. Murray D. (2018). Roles of polyploid/multinucleated giant cancer cells in metastasis and disease relapse following anticancer treatment. Cancers 10:118. 10.3390/cancers10040118 29662021 PMC5923373

[B42] Mirzayans R. Murray D. (2020). Do TUNEL and other apoptosis assays detect cell death in preclinical studies?. Int. J. Mol. Sci. 21:9090. 10.3390/ijms21239090 33260475 PMC7730366

[B43] Nefic H. Handzic I. (2013). The effect of age, sex, and lifestyle factors on micronucleus frequency in peripheral blood lymphocytes of the Bosnian population. Mutat. Res. 753, 1–11. 10.1016/j.mrgentox.2013.03.001 23499242

[B44] Niu N. Mercado-Uribe I. Liu J. (2017). Dedifferentiation into blastomere-like cancer stem cells via formation of polyploid giant cancer cells. Oncogene 36, 4887–4900. 10.1038/onc.2017.72 28436947 PMC5582213

[B45] Niu N. Zhang J. Zhang N. Mercado-Uribe I. Tao F. Han Z.. (2016). Linking genomic reorganization to tumor initiation via the giant cell cycle. Oncogenesis 5:e281. 10.1038/oncsis.2016.75 27991913 PMC5177773

[B46] Pienta K. J. Hammarlund E. U. Austin R. H. Axelrod R. Brown J. S. Amend S. R. (2020a). Cancer cells employ an evolutionarily conserved polyploidization program to resist therapy. Semin. Cancer Biol. 10.1016/j.semcancer.2020.11.016. [Epub ahead of print]. 33276091

[B47] Pienta K. J. Hammarlund E. U. Axelrod R. Amend S. R. Brown J. S. (2020b). Convergent evolution, evolving evolvability, and the origins of lethal cancer. Mol. Cancer Res. 18, 801–810. 10.1158/1541-7786.MCR-19-1158 32234827 PMC7272288

[B48] Pluquet O. Pourtier A. Abbadie C. (2015). The unfolded protein response and cellular senescence. A review in the theme: cellular mechanisms of endoplasmic reticulum stress signaling in health and disease. Am. J. Physiol. Cell Physiol. 308, C415–C425. 10.1152/ajpcell.00334.2014 25540175

[B49] Schmid W. (1975). The micronucleus test. Mutat. Res. 31, 9–15. 10.1016/0165-1161(75)90058-8 48190

[B50] Sears D. A. Udden M. M. (2012). Howell-Jolly bodies: a brief historical review. Am. J. Med. Sci. 343, 407–409. 10.1097/MAJ.0b013e31823020d1 21946828

[B51] Senovilla L. Demont Y. Humeau J. Bloy N. Kroemer G. (2017). Image cytofluorometry for the quantification of ploidy and endoplasmic reticulum stress in cancer cells. Methods Mol. Biol. 1524, 53–64. 10.1007/978-1-4939-6603-5_3 27815895

[B52] Senovilla L. Vitale I. Martins I. Tailler M. Pailleret C. Michaud M.. (2012). An immunosurveillance mechanism controls cancer cell ploidy. Science 337, 1678–1684. 10.1126/science.1224922 23019653

[B53] Shoshani O. Brunner S. F. Yaeger R. Ly P. Nechemia-Arbely Y. Kim D. H.. (2020). Chromothripsis drives the evolution of gene amplification in cancer. Nature 591, 137–141. 10.1038/s41586-020-03064-z 33361815 PMC7933129

[B54] Sommer S. Buraczewska I. Kruszewski M. (2020). Micronucleus assay: the state of art, and future directions. Int. J. Mol. Sci. 21:1534. 10.3390/ijms21041534 32102335 PMC7073234

[B55] Stevens J. B. Abdallah B. Y. Liu G. Ye C. J. Horne S. D. Wang G.. (2011). Diverse system stresses: common mechanisms of chromosome fragmentation. Cell Death Dis. 2:e178. 10.1038/cddis.2011.60 21716293 PMC3169002

[B56] Stevens J. B. Liu G. Bremer S. W. Ye K. J. Xu W. Xu J.. (2007). Mitotic cell death by chromosome fragmentation. Cancer Res. 67, 7686–7694. 10.1158/0008-5472.CAN-07-0472 17699772

[B57] Wang G. Yang Z. Q. Zhang K. (2010). Endoplasmic reticulum stress response in cancer: molecular mechanism and therapeutic potential. Am. J. Transl. Res. 2, 65–74. 20182583 PMC2826823

[B58] Xie Y. Ye S. Zhang J. He M. Dong C. Tu W.. (2016). Protective effect of mild endoplasmic reticulum stress on radiation-induced bystander effects in hepatocyte cells. Sci. Rep. 6:38832. 10.1038/srep38832 27958308 PMC5153638

[B59] Xu B. Wang W. Guo H. Sun Z. Wei Z. Zhang X.. (2014). Oxidative stress preferentially induces a subtype of micronuclei and mediates the genomic instability caused by p53 dysfunction. Mutat. Res. 770, 1–8. 10.1016/j.mrfmmm.2014.08.004 25302047 PMC4186711

[B60] Ye C. J. Sharpe Z. Alemara S. Mackenzie S. Liu G. Abdallah B.. (2019b). Micronuclei cluster and genome chaos: changing the system inheritance. Genes 10:366. 10.3390/genes10050366 31086101 PMC6562739

[B61] Ye C. J. Sharpe Z. Heng H. H. (2020). Origins and consequences of chromosomal instability: from cellular adaptation to genome chaos-mediated system survival. Genes 11:1162. 10.3390/genes11101162 33008067 PMC7601827

[B62] Ye C. J. Stilgenbauer L. Moy A. Liu G. Heng H. H. (2019a). What is karyotype coding and why is genomic topology important for cancer and evolution?. Front. Genet. 10:1082. 10.3389/fgene.2019.01082 31737054 PMC6838208

[B63] Ye J. C. Horne S. Zhang J. Z. Jackson L. Heng H. H. (2021). Therapy induced genome chaos: a novel mechanism of rapid cancer drug resistance. Front. Cell Dev. Biol. 9:676344. 10.3389/fcell.2021.676344 34195196 PMC8237085

[B64] Zanetti M. (2017). Chromosomal chaos silences immune surveillance. Science, New York, NY. 355, 249–250. 10.1126/science.aam5331 28104855

[B65] Zanetti M. Mahadevan N. R. (2012). Cancer. immune surveillance from chromosomal chaos? Science 337, 1616–1617. 10.1126/science.1228464 23019639

[B66] Zhang C. Z. Spektor A. Cornils H. Francis J. M. Jackson E. K. Liu S.. (2015). Chromothripsis from DNA damage in micronuclei. Nature 522, 179–184. 10.1038/nature14493 26017310 PMC4742237

[B67] Zhang S. Mercado-Uribe I. Xing Z. Sun B. Kuang J. Liu J. (2014). Generation of cancer stem-like cells through the formation of polyploid giant cancer cells. Oncogene 33, 116–128. 10.1038/onc.2013.96 23524583 PMC3844126

